# High risks of HIV transmission for men sex worker — a comparison of profile and risk factors of HIV infection between MSM and MSW in China

**DOI:** 10.1186/s12889-022-13264-z

**Published:** 2022-04-29

**Authors:** Maohe Yu, Desheng Song, Tiantian Zhang, Tingting Yao, Yang Chen, Yuanyuan Liu, Elissa Peixoto, Jie Xu, Zhijun Li, Jie Yang, Changping Li, Zhuang Cui

**Affiliations:** 1 STD & AIDS Control and Prevention Section, Tianjin Center for Disease Control and Prevention, Tianjin, China; 2 Department of Epidemiology and Health Statistics, School of Public Health, Tianjin Medical University, Tianjin, 300070 China; 3 National Center for AIDS/STD Control and Prevention, Beijing, China; 4 GAP Program Office of US CDC, Atlanta, GA USA; 5 Tianjin ShenLan Public Health Counseling Service Center, Tianjin, China

**Keywords:** HIV infection, Men who have sex with men, Male sex workers

## Abstract

**Background:**

Although men who have sex with men (MSM) and male sex workers (MSWs) both represent the high-risk groups for the transmission of HIV/AIDS and syphilis, the comparison between them have not yet been well studied in China. We aimed to evaluate the prevalence of HIV among MSM and MSW, and then identify the difference of risk factors of HIV infection.

**Methods:**

A snowball sampling was employed to recruit patrons attending the Tianjin bathhouse from March 2011 to October 2018. A questionnaire covering sociodemographic characteristics, sexual behaviors, HIV-related and HIV awareness was completed by 5166 patrons from all parts of China. Bivariate analyses were done using the Chi-square test to investigate the association between factors and HIV infection among MSM and MSWs. Subsequently, we studied the different impact of risk factors on HIV infections among the two groups using multiple logistic regression with the adjusted odds ratio (aOR) being derived.

**Results:**

From 2011 to 2018, 235 MSWs and 4931 MSM were included into our study. HIV prevalence among the MSWs was 17.8% (95%CI: 13.2% ~ 23.4%) while 6.5% (95%CI: 5.8% ~ 7.2%) for MSM (*P* < 0.01). MSWs tends to be younger (26.50% in MSWs vs. 8.64% in MSM, *P* < 0.05), live alone (84.68% in MSWs vs. 47.98 in MSM, *P* < 0.05), get poor education (41.28% in MSWs vs. 28.45 in MSM, *P* < 0.05), use drug (8.09% in MSWs vs. 0.89% in MSM, *P* < 0.05), have more proportion of always use condom during anal sex (56.50% in MSWs vs. 41.95% in MSM, *P* < 0.05) but less proportion during commercial sex (81.28% in MSWs vs. 98.48% in MSM, *P* < 0.05), access HIV-related health services (65.96% in MSWs vs. 47.80% in MSM, *P* < 0.05) and have a HIV test last year(60.85% in MSWs vs. 41.27% in MSM, *P* < 0.05). The significant associations between risk factors with HIV infection in MSM were not observed in MSWs and vice versa.

**Conclusions:**

High HIV prevalence needs urgent intervention targeting MSWs as a higher susceptible to HIV in comparison to MSM owing to their unique characteristics. The discrepancies of profiles and risk factors between MSM and MSWs should be consider in design and development of strategies.

**Supplementary Information:**

The online version contains supplementary material available at 10.1186/s12889-022-13264-z.

## Background

Men who have sex with men (MSM), identified as a key population, continue to account for 23% of new HIV infections worldwide [1]. In China, the biggest increase in HIV transmission has occurred among MSM, growing from 2.5% of new reported cases in 2006 to 26% in 2014 [2]. HIV prevalence among MSM in Tianjin, the third biggest Chinese city, has hit 12% [3].

Being deeply influenced by Confucianism, the majority of population in China refers to the behavior on having homosexual sex as a violation of moral. To avoid the stigma and discrimination, MSM often marry to women, although they still hold the “sex activities” with other men [4]. Marriage and risk behaviors were frequently mentioned in qualitative research. A study in China found that majority of MSM get married due to pressures like family line [5]. They conceal their sexual orientation [6], and express the willingness to remain contact with their sex partners [4]. This may build a dangerous “bridge” where HIV propagate from MSM to general population.

By contrast, another vulnerable group are MSWs. They sell or exchange sex for money or goods and encompass a very diverse population across and within countries [7]. In Netherlands, an observation study based on clinics indicated 40% of consultations were positive for sexually transmitted diseases (including HIV) within MSWs, while a meta-analysis conducted in 2014 suggested HIV prevalence among MSWs was 15.6% [8, 9]. Besides, a research conducted in Kenya showed that MSWs had the higher risk of HIV infection than MSM (26.3% vs. 12.2%) [10]. The high HIV prevalence within MSWs suggests that they may face unique barriers to accessing healthcare [7], which involved in stigma related to sexuality, gender identity, HIV status, sex worker status and internalized stigma, not only from general population but also MSM [11]. The indicatives of stigma impede them from accessing HIV-related healthcare. Beyond that, there are other biological and behavioral factors, as some qualitative studies suggests, giving rise to the high prevalence within MSWs like risky sexual behaviors and marriage [12]. However, these evidences have not been well-established in China.

Given that, the purposes of our study were to depict the prevalence of HIV infection among MSM and MSWs and then profile the differences in risk factors. Results from this study can be used to develop empirical public health strategies for reducing HIV transmission between MSWs and MSM.

## Methods

### Study setting

Tianjin is one of the major cities in China. Developed transportation makes it the center of male-to-male sexual activities in North of China. In this case, a service station was established at Tianjin MSM bathhouse, where MSM need to pay 20 CNY for entry. The service station refers to two separate rooms set up in gay baths. The service station has three advantages, which are as follows:

Firstly, bathers can be recruited directly in the bathhouse to enter the room for HIV consultation and testing; secondly, the separate room provides a one-to-one private space between the investigator and the participant, which can ensure the highest-level protection of the participant’s privacy; thirdly, the saliva test can also be completed in the service station. In case of a positive result, the bathers will go through a confirming test as soon as possible and will receive professional interpretation on the results.

In this research, after rapid HIV test was finished, an anonymous questionnaire was administered by trained staff from the AIDS integrated intervention service center. MSM would be included our cross-sectional analysis if they met the following criteria: (1) ≥18 years, (2) identified as male when born, (3) have intercourse with another men, (4) signed up the informed consent and (5) are able to understand the privacy issues and service involved in the research process.

### Sampling

Because of the particularity of MSW and MSM, a snowball sampling was employed. In this case, our research staff have an office in the bathhouse. Every MSW or MSM coming to the office for consulting may leave their fingerprints. Privacy of the respondents would be fully guaranteed. All participants in the research are anonymous. They do not need to provide their real names and ID cards; only need to provide a pseudonym or nickname, so that all researchers, including investigators, will not know the real name of the participant. Those who participated in the survey must be compensated and benefited for their time. The benefit is that they can get free AIDS counseling and testing since they usually enter the bathhouse to find sexual partners or seek homosexual sex. The fingerprint information was collected with the informed consent of the investigator and was only used to identify the repeated testing of the respondent. Certainly, if the respondent does not want to register fingerprints, his wishes would be respected, but he would be excluded from the study. In fact, this situation is very rare. Information on fingerprint and questionnaire obtained in the survey, used only for this project, would be strictly stored in AIDS prevention and management system (APMS) developed for the study and each investigator has undergone uniform training and signed a data confidentiality agreement.

Besides, the bathers would be also encouraged to bring their sex partners for HIV counseling and testing. At the same time, they would also receive free condoms and lubricants after completing the investigation which thus prompt them better protects for themselves and others during sex.

### Measurement

Private interviews were performed by investigators with long-time experience working with this population. Each of them had been trained by the study’s key members. Interviews were conducted to obtain desired information from 42 questions grouped into demographics, knowledge about HIV, sexual behaviors, substance abuse, STIs, self-reported risk of infection and the uptake of health service. Social-demographic information included year of birth, marital status, household register, nationality and education; additionally, the living arrangement was derived from marital status with those single or divorced/widowed patrons referred to be living alone while the people married or in cohabitation as living with a partner (male or female). HIV aware was evaluated by eight basic knowledge of AIDS prevention in China; sexual behaviors consisted of age of first sexual intercourse with men, anal sex, condom use, commercial sex and so forth. Five questions for the staff need to be finished after a subject answered all the questions and we got the subjects’ HIV test outcome. Whether the subject is MSW is one of the five questions above. Other questions were the rapid test outcomes and confirmed results of AIDS.

The questionnaire is presented in Supplement.

### Data collection

When coming into our office, those patrons would receive information regarding why, how, and when to test, and were asked if they wanted to join the study. If yes, their fingerprint would be collected and an informed consent assigned; otherwise, they were remobilized. Then, a face-to-face HIV counseling with intra-speaking started and part of questionnaire related participants was filled out. Next, a rapid test was conducted. Within 20 min of waiting for the results, our investigators would conduct a risk assessment based on the completion of the questionnaire. If the test results were negative, it would be told; if positive, post-exposure advice would be given. Finally, participants would be given free condoms and lubricants and encouraged to mobilize their partners to test as well.

### HIV test

According to their selection, saliva or blood was collected. Patrons’ oral mucosal exudate test (Mano Biopharmaceutical Co., Ltd., Beijing, China) was used for the former and a blood rapid detection reagent (Wan Fu Biotechnology Co., Ltd., Guangzhou, China) for the latter. In addition, 5 ml of the blood sample was collected from those who get any positive tests above. Then, the blood sample was sent to district CDCs to perform enzyme-linked immunosorbent (ELISA; Wan Tai Biological Pharmaceutical Co., Ltd., Beijing, China). Confirmation of HIV infection was conducted by a Western blot (WB) assay (MP Biomedical Asia Pacific Pte Ltd., Singapore).

### Data analysis

To understand the true characters around their seroconversion, we collected the latest record on whether they persist HIV-negative or the exact record of HIV-positive, which comprises data included in our analysis. All the categorical data was presented as n (%). *P* values< 0.05 for a two-tailed test was referred to be statistically significant.

To compare the distribution of characteristics in MSWs and MSM, we used the Chi-square test. The same test was also used to investigate the association between characteristics and HIV infection in the two groups.

Multivariable Logistic regression was used to quantify the increased risk in HIV infection among MSWs who engage in male sex industry in comparison with MSM, adjusted by marital status, the highest education, anal sex, group sex, commercial sex and having sex with women in the past 6 months along with corresponding frequencies of condom use, uptake of HIV-related health services and HIV test last year. These adjusted risk factors were either with significantly distribution between MSM and MSWs or well-studied variables independently related to HIV infection in previous studies. The final model was derived using stepwise selection from full model. Further, we conducted the same analyses in MSWs and MSM, respectively.

This study was reviewed and approved by the Institutional Review Board of the National Center for AIDS / STD Prevention and Control of the Chinese Centers for Disease Control and Prevention [IRB approve number: X130205267] and sponsored by the President’s Emergency Plan for AIDS Relief (PEPFAR) and the Humanities and Social Science Fund of the Ministry of Education, China. All participants signed an informed consent form before the survey started.

All the statistical analysis was performed with SAS 9.4 (Cary, NC, USA).

## Results

From 2011 to 2018, 235 MSWs and 4931 MSM were included into our study and 363 bathers were identified as HIV positive. Of MSWs, the prevalence of HIV infection was 17.9% (42/235, 95%CI:13.2% ~ 23.4%). By contrast, the prevalence was 6.5% in MSM (320/4931, 95%CI:5.8% ~ 7.2%).

**Fig. 1:**
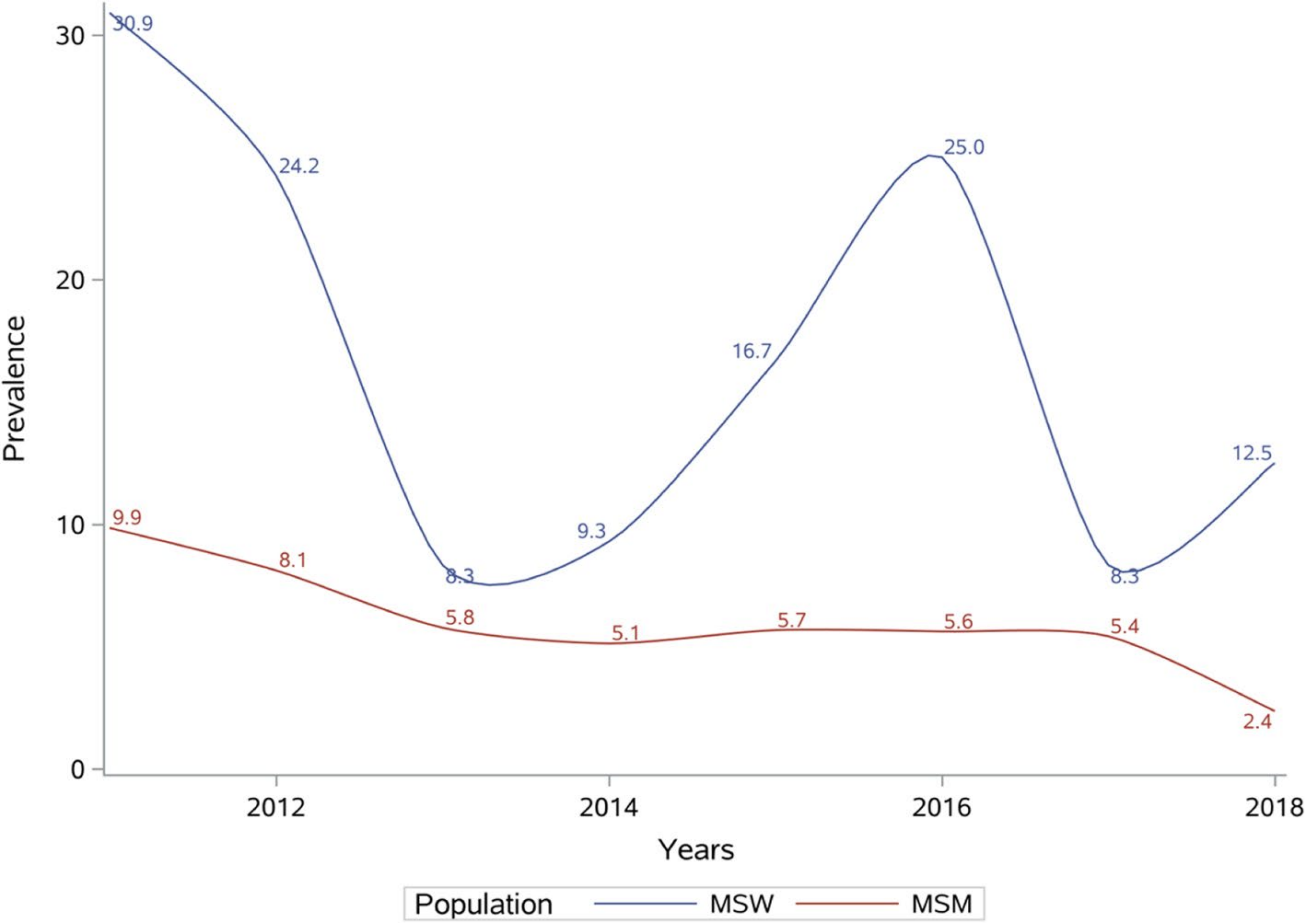
The prevalence of HIV infection among MSWs and MSM from 2011 to 2018

As depicted by Fig. 1**,** during the 8 years, the nonlinear spline showed a decreasing trend in prevalence in MSM. However, a “down-up” fluctuation could be observed in MSWs in terms of prevalence of acquired HIV. It is worth mentioning that the prevalence of HIV infection in MSWs was always higher than that in MSM’s. Until 2018, the prevalence of HIV infection has fell below 3% while corresponding number continued to be higher than 10% in MSWs.

Compared with MSM, there were significant differences in some characteristics such as marital status, highest education, districts, sites for seeing partners, HIV awareness, unprotected sex behaviors, having sex with women, drug use, uptake of health service and test last year (*P* < 0.05), as presented by Table 1.

**Table 1:** The distribution of characteristics in MSM and MSWs

Factors	Population		Total	***P***
	MSWs (***N*** = 235)	MSM (***N*** = 4931)		
Age				< 0.0001
18 ~ 24	62 (26.50)	425 (8.64)	487 (9.43)	
25 ~ 44	158 (67.52)	3070 (62.42)	3228 (62.49)	
45~	14 (5.98)	1423 (28.93)	1437 (27.82)	
Marital status				< 0.0001
UWD^a^	199 (84.68)	2366 (47.98)	2565 (49.74)	
MC	35 (14.89)	2555 (51.82)	2592 (50.26)	
Census				< 0.0001
Local	55 (23.40)	2078 (42.14)	2134 (41.38)	
Outsider	179 (76.17)	2836 (57.51)	3016 (58.48)	
Foreigner	0 (0.00)	7 (0.14)	7 (0.14)	
Highest education				< 0.0001
Junior school	97 (41.28)	1403 (28.45)	1502 (29.13)	
High school	100 (42.55)	1921 (38.96)	2021 (39.20)	
College	37 (15.74)	1596 (32.37)	1633 (31.67)	
Have anal sex last 6 m^b^				< 0.0001
Yes	223 (94.89)	4500 (91.26)	4725 (91.46)	
No	12 (5.11)	429 (8.70)	441 (8.54)	
Frequency of condom use during anal sex				< 0.0001
Never	5 (2.13)	166 (3.37)	171 (3.31)	
Sometimes	97 (41.28)	2697 (54.69)	2796 (54.10)	
Always	133 (56.60)	2068 (41.94)	2201 (42.59)	
Have commercial sex last 6 m				< 0.0001
Yes	163 (69.36)	296 (6.00)	459 (8.88)	
No	72 (30.64)	4634 (93.98)	4708 (91.12)	
Frequency of condom use during commercial sex				< 0.0001
Never	5 (2.13)	10 (0.20)	15 (0.29)	
Sometimes	39 (16.60)	64 (1.30)	103 (1.99)	
Always	191 (81.28)	4856 (98.48)	5049 (97.72)	
Have sex with women				< 0.0001
Yes	44 (18.72)	1790 (36.30)	1834 (35.49)	
No	191 (81.28)	3141 (63.70)	3334 (64.51)	
Frequency of condom use during sex with women				< 0.0001
Never	20 (8.51)	1276 (25.88)	1296 (25.09)	
Sometimes	7 (2.98)	264 (5.35)	271 (5.25)	
Always	207 (88.09)	3390 (68.75)	3599 (69.67)	
Drug use				< 0.0001
Yes	19 (8.09)	44 (0.89)	63 (1.22)	
No	216 (91.91)	4885 (99.07)	5103 (98.78)	
Uptake of HIV-related service				< 0.0001
Yes	155 (65.96)	2357 (47.80)	2514 (48.65)	
No	80 (34.04)	2573 (52.18)	2653 (51.35)	
Have a HIV test last year				< 0.0001
Yes	143 (60.85)	2035 (41.27)	2179 (42.16)	
No	92 (39.15)	2896 (58.73)	2989 (57.84)	
Chance of HIV acquired				< 0.0001
Low	23 (9.79)	141 (2.86)	165 (3.19)	
Medium	79 (33.62)	1614 (32.73)	1694 (32.80)	
High	133 (56.60)	3173 (64.35)	3306 (64.01)	
Have group sex last 6 m				< 0.0001
Yes	57 (24.26)	645 (13.08)	702 (13.59)	
No	178 (75.74)	4284 (86.88)	4464 (86.41)	
Frequency of condom use during group sex				< 0.0001
Never	1 (0.43)	36 (0.73)	37 (0.72)	
Sometimes	15 (6.38)	252 (5.11)	267 (5.17)	
Always	219 (93.19)	4641 (94.12)	4862 (94.12)	
HIV test result				< 0.0001
Positive	42 (17.87)	320 (6.49)	363 (7.02)	
Negative	193 (82.13)	4611 (93.51)	4805 (92.98)	

*P* < 0.0001

^a^*UWD* Unmarried, Windowed and Divorced, *MC* Married and Cohabitation

^b^6 m represent 6 months

Engaging in male sex industry was associated with increased risk of HIV infection in multivariable analysis with aOR (3.38, 95%CI:2.14 ~ 5.32). In detail, in multivariable analysis using variable selection, we adjusted those variables, which were accepted as risk factors, including marital status, age, highest education, self-reported chance of HIV acquired, and having sex with women.

Univariate analysis showed that marital status(*P* < 0.0001), highest education (*P* < 0.0001), anal sex(*P* < 0.0001), commercial sex(*P* < 0.0001), group sex(*P* < 0.0001) and the frequencies of condom use (*P* < 0.0001, respectively), uptake of HIV-related service (*P* = 0.0067) and HIV test last year (*P* = 0.0041) were significantly associated with HIV infection. Thus, we included those variables into multiple logistic models and then derived aORs (95% CI) of factors in MSM and MSWs, respectively. Tables 2 and 3 showed the different association of some variables on HIV infection in the two groups. As suggested by multiple variables analysis using stepwise selection, there exist some variables with aORs significantly bigger than 1 in MSM while the counterpart in MSWs were not such as age, marital status, the highest education, chance of HIV acquired, having a HIV test last year and so on.

**Table 2:** The associations between focused factors and HIV within MSM

Factors	n(%)^a^	Crude OR(95% CI)	Adjusted OR(95% CI)
Age			
18 ~ 24	93 (5.8)	0.59 (0.41 ~ 0.83)	0.29 (0.19 ~ 0.45)
25 ~ 44	174 (6.3)	0.63 (0.46 ~ 0.87)	0.40 (0.31 ~ 0.52)
> = 45^b^	53 (9.6)	–	–
Marital status^d^			
UWD	260 (11.0)	5.13 (3.85 ~ 6.84)	13.93 (10.03 ~ 19.35)
MC^b^	60 (2.3)	–	–
Highest education			
Junior school	130 (9.3)	0.62 (0.49 ~ 0.79)	2.09 (1.57 ~ 2.76)
High school	99 (5.2)	0.90 (0.67 ~ 1.20)	0.96 (0.72 ~ 1.28)
College^b^	91 (5.7)	–	–
Have anal sex last 6 m^c^			
Yes	300 (6.7)	1.46 (0.92 ~ 2.32)	
No^b^	20 (4.7)	–	–
Have group sex last 6 m^c^			
Yes	45 (7.0)	1.09 (0.79 ~ 1.52)	
No^b^	275 (6.4)	–	–
Have commercial sex last 6 m^c^			
Yes	16 (5.4)	0.81 (0.49 ~ 1.37)	
No^b^	304 (6.6)	–	–
Frequency of condom use during anal sex^c^			
Never	21 (12.7)	3.55 (2.14 ~ 5.91)	
Sometime	218 (8.1)	2.16 (1.66 ~ 2.80)	
Always^b^	81 (3.9)	–	–
Frequency of condom use during group sex^c^			
Never	9 (25.00)	5.00 (2.33 ~ 10.73)	
Sometime	21 (8.3)	1.36 (0.86 ~ 2.17)	
Always^b^	290 (6.2)	–	–
Frequency of condom use during commercial sex^c^			
Never	3 (30.0)	6.29 (1.62 ~ 24.42)	
Sometime	7 (10.9)	1.80 (0.82 ~ 3.98)	
Always^b^	310 (6.4)	–	–
Frequency of condom use during sex with women^c^			
Never	69 (5.4)	0.77 (0.58 ~ 1.01)	
Sometime	16 (6.1)	0.87 (0.51 ~ 1.46)	
Always^b^	235 (6.9)	–	–
Uptake of HIV-related service^c^			
Yes	134 (5.7)	0.77 (0.62 ~ 0.97)	
No^b^	186 (7.2)	–	–
Chance of HIV acquired			
High	20 (14.2)	2.92 (1.78 ~ 4.81)	2.96 (1.78 ~ 4.94)
Medium	130 (8.1)	1.55 (1.22 ~ 1.96)	1.82 (1.45 ~ 2.29)
Low^b^	170 (5.4)	–	–
Have a HIV test last year			
Yes	101 (5.0)	0.64 (0.50 ~ 0.61)	0.63 (0.50 ~ 0.79)
No^b^	219 (7.6)	–	–

^a^N of participants who were infected with HIV

^b^Reference level

^c^Not included in the final model

^d^*UWD* Unmarried, Windowed and Divorced, *MC* Married and Cohabitation

**Table 3:** The associations between focused factors and HIV within MSWs

Factors	n(%)^a^	Crude OR(95% CI)	Adjusted OR(95% CI)
Age^c^			
18 ~ 24	28 (16.4)	0.64 (0.31 ~ 1.33)	–
25 ~ 44^b^	14 (23.3)	–	–
> = 45		–	–
Marital status^cd^			
UWD	39 (19.6)	2.60 (0.76 ~ 8.93)	–
MC^b^	3 (8.6)		–
Highest education^c^			
Junior school	16 (16.5)	0.85 (0.32 ~ 2.26)	–
High school	19 (19.0)	1.01 (0.38 ~ 2.63)	–
College^b^	7 (18.9)		
Have anal sex last 6 m^c^			
Yes	41 (18.4)	2.48 (0.31 ~ 19.73)	–
No^b^	1 (8.3)		
Have group sex last 6 m^c^			
Yes	5 (8.8)	0.37 (0.14 ~ 0.98)	–
No^b^	37 (20.8)		
Have commercial sex last 6 m^c^			
Yes	24 (14.7)	0.52 (0.26 ~ 1.03)	–
No^b^	18 (25.0)		
Frequency of condom use during anal sex^c^			
Never	1 (20.0)	1.41 (0.15 ~ 13.30)	–
Sometime	21 (21.6)	1.56 (0.79 ~ 3.08)	–
Always^b^	20 (15.0)		
Frequency of condom use during group sex^c^			
Never	–	–	–
Sometime	4 (26.7)	1.73 (0.52 ~ 5.73)	–
Always^b^	38 (17.4)		
Frequency of condom use during commercial sex^c^			
Never	2 (40.0)	3.19 (0.51 ~ 19.86)	–
Sometime	7 (17.9)	1.05 (0.43 ~ 2.58)	–
Always^b^	33 (17.3)		
Frequency of condom use during sex with women^c^			
Never	5 (25.0)	1.64 (0.56 ~ 4.80)	–
Sometime	2 (28.6)	2.00 (0.37 ~ 10.54)	–
Always^b^	35 (16.9)		
Uptake of HIV-related service^c^			
Yes	29 (18.7)	1.19 (0.58 ~ 2.43)	–
No^b^	13 (16.3)		
Chance of HIV acquired^c^			
High	6 (26.1)	1.99 (0.70 ~ 5.67)	–
Medium	16 (20.3)	1.44 (0.69 ~ 2.97)	–
Low^b^	20 (15.0)		
Have a HIV test last year^c^			
Yes	26 (18.2)	1.06 (0.53 ~ 2.10)	–
No^b^	16 (17.4)		

^a^N of participants who were infected with HIV

^b^Reference level

^c^Not included in the final model

^d^*UWD* Unmarried, Widowed or Divorced, *MC* Married or Cohabitation

## Discussion

Compared with MSM, MSWs tend to be younger, have lower education and most of them were not locals from Tianjin. These characteristics reflected some noteworthy profiles including the low-income level of the MSW population, sex services for goods, and high mobility, which have become obstacles to their accessibility and intervention [13].

In our study, within MSM, the prevalence of HIV infection was 6.5% while 17.9% among MSWs, which was similar to the research in Shanghai [14] and Shenzhen [15]. Undoubtedly, MSWs have become a non-negligible group for HIV infection in China.

From 2011 to 2018, the comparison between the downward trend of HIV infection among MSM, which has parallel results with Cui [16] and the fluctuation among MSW population indicated, to a certain extent, that the strategy for MSM is not applicable to MSWs. The reasons for this phenomenon might be as follows: firstly, due to the health promotion and education from the station set up in the bathhouse, MSM has increased their awareness of protection and reduced dangerous sexual behaviors; secondly, MSWs tend to be younger, have lower education in comparison with MSM [17] and they usually sell sex for money and goods, so changes in cognition may not be necessarily reflected in actions; thirdly, during sex with others, male sex workers are usually passive, as they need to meet the needs from customers.

The test rate among MSWs was significantly higher than MSM. In terms of sexual behavior, the frequencies of using condoms among MSWs was not lower than MSM in high-risk sexual behaviors (homosexual anal intercourse, group sex and commercial sex). Nevertheless, the prevalence of HIV infection for MSWs was still more than twice that of MSM. There may be some reasons for this. Firstly, there might not be use of condom in the entire process of sexual behavior. Due to the professional requirements of MSWs, they would try their best to meet their customers’ sexual desires. Therefore, though MSWs have a better grasp of HIV knowledge than MSM, unprotected sexual behavior still occurs; secondly, the frequency of drug use in MSWs was significantly higher than MSM. According to related research [18], drugs are positively correlated with HIV infection. As a psychological booster, it could obscure the consciousness of MSWs and clients, causing them to have no awareness of condom use during sex with their customers. This undoubtedly greatly increases the risk of HIV infection in MSWs. Finally, the information we collected about condom use was self-reported by MSM and MSWs, which may be overestimated due to social-expectation bias.

The differences in characteristics may lead to differences in association between risk factors and HIV infections. As suggested by Table 2 and Table 3, the associations were different in MSWs and MSM. The UWD group has higher risk than MC, which is in line with a previous study in China. While that association was observed in MSM, it was not in MSWs. This may be due to the fact that most of MSWs were youngers, and we cannot get enough examples aged > 30 just like in MSM. What’s more, due to characteristic of MSWs, even though some got married, they often don’t stop dangerous sex behaviors. Similarly, no matter what the highest education was, they must continue to meet the needs from clients to exchange money and goods. Therefore, although they were likely to have more frequencies and more uptake of HIV-related service than MSM, similar results could not be found.

Our study had several strengths. Firstly, to our knowledge, this bath-based study was the one of the largest observational studies collecting more than 5000 MSM in China. Moreover, we focus on MSWs, the understudied population in MSM in China. The present study disclosed a remarkably higher risk of HIV infection among MSWs, indicating urgent measures should be considered on the vulnerable population in grey areas. Secondly, diversity of MSM was recruited in the present study. The age of patrons coming into the bathhouse ranges from 18 to 80 years old, the hukou of participants covers most regions of China and the variety of ways of recruit (bars, clubhouses, peer referral and internet) are adopted. Feasibility, authenticity, and diversity can be guaranteed. Finally, our study explored the discrepancy in demographic and sex behavioral features between MSM and MSWs, which could provide valuable information for implementation of targeted HIV/AIDS health and education interventions among MSWs. Our study also had some limitations. A snowball sampling was used for accessing MSM or MSWs, which can cause some selection bias. But in the context of the Tianjin Bathhouse, we could obtain all kinds of information from patrons around China. The data of this study was obtained through interview questionnaires and was inevitably subject to information bias due to particularities of MSWs. However, our investigators themselves are also MSM, along with that survey was conducted on a one-to-one basis using words and expressions from their cultural circles. Thus, the bias has been minimized. Although the questionnaire contains information involved in demographics, behaviors and services, there were still some issues not mentioned such as their posture during sex, which needs to be studied further.

In conclusion, there were some differences in characteristics between MSWs and MSM and the amounts of effects of risk factors on HIV infection were also different. These disagreements should be considered in design of strategies targeting to MSWs and MSM, respectively.

## Supplementary Information

**Additional file 1.** Questionnaire for the study. This is the questionnaire for the study which participants and staffs completed.

## Data Availability

The datasets used and analyzed during the current study are available from the corresponding author on reasonable request.

## Abbreviations

MSM: Men who have sex with men
MSW: Male sex worker
