# Supplementary material for: High risks of HIV transmission for men sex worker — a comparison of profile and risk factors of HIV infection between MSM and MSW in China
Source: BMC Public Health. 2022 Apr 29;22:858. doi: 10.1186/s12889-022-13264-z (PMC9052596; doi:10.1186/s12889-022-13264-z)
Supplement: Supplementary file 1 — Additional file 1. Questionnaire for the study. This is the questionnaire for the study which participants and staffs completed. [file 12889_2022_13264_MOESM1_ESM.docx]

Survey Questionnaire

A01 Place: Tianjin
A02 Type: YMSM

A03 GB code of local administrative divisions: 120000

A04 No.□□□□(0001—9999)

A05 Fill in date □□□□year □□month □□day

Hello, my name is XXX, from Tianjin Shenlan CBO. We are surveying to investigate people's knowledge and behavior about some health problems. Please feel assured that this investigation is anonymous and we will keep your answers confidential. We hope your answers are true. The survey will take you about 10 minutes. I can help you at the end of the survey (for example, you can ask some health questions, I will try to answer). I hope you will support our work. Thank you! Interviewee: have you participated in this survey recently? If the answer is "yes", the visit will end.

**Part 1 Demographic information**

B01 Year of birth [fill in the blank]

______

B02 Marital status [single choice question]

(1)Unmarried

(2)In marriage

(3)Cohabitation

(4)Divorced or widowed

(5)Others (Please specify:_____)

B03 Location of household registration [single choice question]

(1)Tianjin

(2)Other province (Please specify:_____)

(3)Foreign (Please specify:______) (Jump to B05)

B04 Nationality [fill in the blank]

______

B05 How long have you lived in Tianjin? [single choice question]

(1)<3 Months

(2)3~6 Months

(3)7~12 Months

(4)1~2 Years

(5)2 Years or more

B06 Education level [single choice question]

(1)Illiterate

(2)Primary school

(3)Junior high school

(4)Senior high or secondary school

(5)College

(6)Bachelor’s degree

(7)Master’s degree or above

B07 What is your sexual orientation? [single choice question]

(1)Homosexuality

(2)Heterosexuality

(3)Bisexuality

(4)Undetermined

B08 What is your most important route to find a male sexual partner? [single choice question]

(1)Bar/Dance hall

(2)Tea House/Club

(3)Bath

(4)Park/Public toilet/Lawn

(5)Internet/Dating software

(6)Other places (Please specify:_____)

B09 What is your job? [single choice question]

(1)Student

(2)Teacher

(3)Worker of the service industry

(4)Unemployed

(5)Male sex worker

(6)Others (Please specify:_____)

**Part 2 HIV/AIDS Knowledge**

C01 Is AIDS a serious and incurable infectious disease? [single choice question]

(1)Yes

(2)No

(3)I don't know

C02 Are men who have sex with men (MSM) the most severe population affected by AIDS in China? [single choice question]

(1)Yes

(2)No

(3)I don't know

C03 Can we judge whether a person is infected with AIDS by his appearance? [single choice question]

(1)Yes

(2)No

(3)I don't know

C04 Does infection with other STDs increase the risk of HIV infection? [single choice question]

(1)Yes

(2)No

(3)I don't know

C05 Can the use of condoms reduce the risk of HIV infection and transmission? [single choice question]

(1)Yes

(2)No

(3)I don't know

C06 Can the use of new drugs (methamphetamine, ecstasy, ketamine, etc.) increase the risk of HIV infection? [single choice question]

(1)Yes

(2)No

(3)I don't know

C07 Should a person seek HIV testing and counseling actively after high-risk behaviors (needle sharing, drug use/unsafe sex, etc.)? [single choice question]

(1)Yes

(2)No

(3)I don't know

C08 Does a person need to bear legal responsibility for the intentional transmission of AIDS? [single choice question]

(1)Yes

(2)No

(3)I don't know

**Part 3 Sexual behaviors**

D001 The age when you first had sex with a man [fill in the blank]

______

D01 In the past six months, have you ever had sex with a male? [single choice question]

(1)Yes

(2)No

D011 In the past six months, how many people of the same sex have you had anal sex with? [fill in the blank]

______

D02 In the past week, how many times have you had anal sex with a male? [fill in the blank]

______

D021 In the past week, how many people of the same sex have you had anal sex with? [fill in the blank]

______

D03 Did you use condoms the last time you had sex with a male? [single choice question]

(1)Yes

(2)No

D04 In the past six months, how often do you use condoms when you had anal sex with males? [single choice question]

(1)Never

(2)Sometimes

(3)Always

E01 In the past six months, have you ever had commercial sex with a male? [single choice question]

(1)Yes

(2)No (Jump to F01)

E02 In the past six months, how often do you use condoms in commercial sex? [single choice question]

(1)Never

(2)Sometimes

(3)Always

E03 Did you use condoms the last time you had commercial sex with a male? [single choice question]

(1)Yes

(2)No

F01 In the past six months, have you ever had sex with a female? [single choice question]

(1)Yes

(2)No (Jump to G01)

F02 In the past six months, how often do you use condoms when you had sex with a female? [single choice question]

(1)Never

(2)Sometimes

(3)Always

F03 Did you use condoms the last time you had sex with a female? [single choice question]

(1)Yes

(2)No

**Part 4 Addictive substance**

G01 Have you ever used drugs (including meth, ketamine, and other new drugs)? [single choice question]

(1)Yes

(2)No (Jump to G05)

G02 Have you ever injected drugs? [single choice question]

(1)Yes

(2)No (Jump to G05)

G03 Have you ever shared needles with others? [single choice question]

(1)Yes

(2)No (Jump to G05)

G04 When injecting drugs in the past six months, how often do you share needles with others? [single choice question]

(1)Never

(2)Sometimes

(3) Always

G05 Have your ever used recreational drugs (any recreational drugs like rush poppers, methamphetamine or 0 number) [single choice question]

(1)Yes

(2)No

G06 What kind of recreational drugs have you used? [multiple choice question]

(1)Rush poppers

(2)Methamphetamine

(3)0 number capsule (also known as 5-MeO-DiPT, Foxy, and Foxy Methoxy)

(4)Others (Please specify:_____)

G07 Do you smoke currently? [single choice question]

(1)Yes

(2)No

G08 Do you drink currently? [single choice question]

(1)Yes

(2)No (Jump to H01)

G09 What kind of wine do you drink? [multiple choice question]

(1)Beer

(2)Red wine

(3)White wine

(4)Others (Please specify:_____)

**Part 5 Sexually transmitted diseases**

H01 In the past year, have you ever been diagnosed with a sexually transmitted disease? [single choice question]

(1)Yes

(2)No (Jump to I01)

H02 In the past year, what sexually transmitted disease have you been diagnosed? [multiple choice question]

(1)Gonorrhea

(2)Syphilis

(3)Chlamydia trachomatis infection of reproductive tract

(4)Condyloma acuminatum

(5)Genital herpes

(6)Others (Please specify:_____)

**Part 7 Utilization of services**

In the past 6 months, have you received the following services for HIV prevention?

I01 Condom promotion and provide/AIDS Counseling [single choice question]

(1)Yes

(2)No

I02 Community medication maintenance treatment/cleaning needle supply or exchange [single choice question]

(1)Yes

(2)No

I03 Peer education [single choice question]

(1)Yes

(2)No

**Part 8 HIV testing**

J01In the past year, have you had HIV test? [single choice question]

(1)Yes

(2)No (Jump to J03)

J02Do you know the test results yourself? [single choice question]

(1)Yes

(2)No

J03 When was your last test? (If you have never done it before, leave it blank, end) [fill in the blank]

Year _______

Month _______

J04 What was the result of your last test? [single choice question]

(1)Negative

(2)Positive

(3)I don't know

**Part 9 PrEP and PEP**

K01 Have you ever heard of pre-exposure prophylaxis (PrEP)? [single choice question]

(1)Yes

(2)No (Jump to K05)

K02 Would you like to use pre-exposure prophylaxis (PrEP)? [single choice question]

(1)Yes (Jump to K04)

(2)No

K03 Why you are unwilling to use pre-exposure prophylaxis (PrEP)? [multiple choice question]

(1)High price of PrEP

(2)Worry about side effects

(3)Worry about the prevention effectiveness

(4)Can’t take medicine every day

(5)Use condoms consistently

(6)Have a regular sex partner

(7)Others (Please specify:_____)

K04 What is the highest price you can afford for preventive medicine (the current price is expected to be 1980 yuan/month)? [fill in the blank]

_____

K05 Have you ever heard of post-exposure prophylaxis (PEP)? [single choice question]

(1)Yes

(2)No

K06 Do you know where to buy post-exposure prophylaxis (PEP)? [single choice question]

(1)I don’t know

(2)Sunlight Male Hospital of Tianjin

(3)Shenlan Community-Based Organization

(4)Tianjin Centers for Disease Control and Prevention (CDC)

(5)Other organizations____________

**Registration is over, thank you!
-------------------------------------------------------------------------------------------------------------------**

**The following are filled out by the staff:**

**HIV screening test results:**

T01 Whether blood was collected in this investigation? [single choice question]

(1)Yes

(2)No

T02a Whether it has been tested positive for HIV in the past? [single choice question]

(1)Yes

(2)No (Jump to T03)

T02b When is the earliest confirmation test positive? [fill in the blank]

Year _______

Month _______

**T03 HIV antibody test results:**

T03a What is the first ELTSA screening result? [single choice question]

(1)Positive

(2)Negative (Jump to T04)

T03b What is the second ELISA reexamination result? [single choice question]

(1)Positive

(2)Negative

T03c What is the confirmatory test result? [single choice question]

(1)Positive

(2)Negative

(3)Suspicious

(4)Not detected

**T04 Syphilis test results:**

T04a What is the ELTSA result? [single choice question]

(1)Positive

(2)Negative (skip to T05)

T04b What is the RPR/TRUST test result? [single choice question]

(1)Positive

(2)Negative

**T05 HCV test results:**

T05a What is the first ELISA screening result? [single choice question]

(1)Positive

(2)Negative (End)

T05b What is the second ELISA reexamination result? [single choice question]

(1)Positive

(2)Negative
